# A systematic review and meta-analysis of the role of Doppler ultrasonography of the superior mesenteric artery in detecting neonates at risk of necrotizing enterocolitis

**DOI:** 10.1007/s00247-023-05695-6

**Published:** 2023-06-13

**Authors:** Dimitrios Rallis, Konstantina Kapetaniou, Pavlos Machas, Foteini Balomenou, Vasileios Giapros, Efstratios Saliakellis

**Affiliations:** 1 Neonatal Intensive Care Unit, University of Ioannina, School of Medicine, Stavrou Niarchou Avenue, 45500 Ioannina, Greece; 2 Department of Pediatrics, University of Ioannina, School of Medicine, Ioannina, Greece; 3 Department of Pediatrics, Democritus University of Thrace, Alexandroupolis, Greece

**Keywords:** Doppler ultrasonography, End-diastolic velocity, Ischemia, Necrotizing enterocolitis, Neonates, Peak systolic velocity, Perfusion

## Abstract

The role of postnatal Doppler measurements of the superior mesenteric artery (SMA) in detecting neonates at risk of necrotizing enterocolitis (NEC) remains uncertain; therefore, we systematically reviewed and meta-analyzed the existing evidence regarding the usefulness of SMA Doppler measurements in detecting neonates at risk for NEC. We used the Preferred Reporting Items for Systematic reviews and Meta-Analyses guidelines, and we included studies which reported the following Doppler ultrasonography indices: peak systolic velocity, end-diastolic velocity, time average mean velocity, differential velocity, pulsatility index (PI) and resistive index. Eight studies were eligible for inclusion in the meta-analysis. Evidence suggested that, during the first postnatal day, neonates who developed NEC had a significantly higher peak systolic velocity (mean difference of 2.65 cm/s (95% confidence interval [CI] 1.23, 4.06, overall effect *Z*=3.66, *P*<0.001)), higher PI (mean difference of 1.52 (95% CI 0.00, 3.04, *Z*=1.96, *P*=0.05)) and higher resistive index (mean difference of 1.09 (95% CI 0.59, 1.60, *Z*=4.24, *P*<0.001)), compared to neonates who did not develop NEC. However, our findings do not support a strong association between the Doppler ultrasound indices and development of NEC at the time of disease onset. This meta-analysis suggests that first postnatal day SMA Doppler parameters, namely peak systolic velocity, PI and resistive index, are higher in neonates who develop NEC. On the other hand, the aforementioned indices are of uncertain significance once the diagnosis of NEC has been established.

**Graphical abstract:**

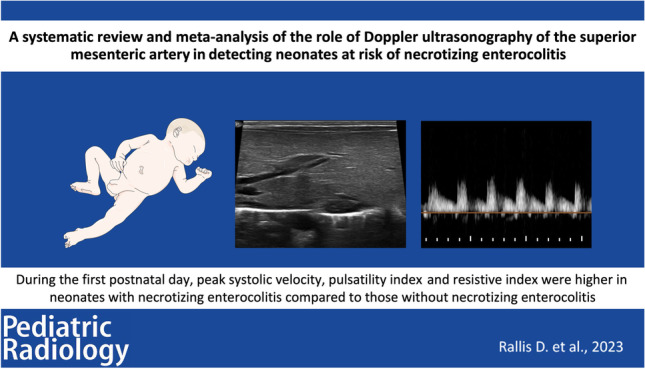

**Supplementary Information:**

Supplementary material is available at 10.1007/s00247-023-05695-6.

## Introduction

Necrotizing enterocolitis (NEC) is a devastating gastrointestinal disease in neonates and is characterized by intestinal injury, systemic inflammation and multisystem organ failure [1]. Extremely preterm or low birth weight neonates, neonates with intrauterine growth restriction, term neonates who were exposed to a hypoxic, ischemic, or infective insult or those neonates with abnormal antenatal Doppler indices in the umbilical artery are at increased risk of developing NEC [2].

Prenatal placental failure is a risk factor for antenatal NEC due to increased placental vascular resistance which results in a reduction of end-diastolic blood flow in the umbilical arteries, ultimately progressing to absent or reversed end-diastolic flow [3]. Fetal adaptation to chronic hypoxia involves preferential shunting of blood to the brain at the expense of the splanchnic circulation [4]. The superior mesenteric artery (SMA) is one of the three major visceral arteries arising from the abdominal aorta and is the main blood supply to the small intestine and a portion of the large intestine [5]. Although prenatal SMA blood flow is diminished, manifesting as high resistance, it increases rapidly after birth because of the change in fetal circulation [5–7]. Doppler ultrasound may be a valuable tool in monitoring disease progression as it enables the detection of changes in intestinal perfusion before severe damage to the intestinal epithelium occurs [8, 9]. Recent experimental and clinical studies have shown that abnormal perfusion in the splanchnic circulation, particularly in the SMA, may have a role in the development of NEC in newborns [10–13]. Hence, research has been conducted on the value of Doppler ultrasound for the prediction, early diagnosis and evaluation of NEC progression in the abovementioned group of patients.

To date, studies have mainly focused on the role of prenatal examination of umbilical artery Doppler indices in the evaluation of the risk of NEC; however, the evidence regarding the role of postnatal measurement of SMA Doppler indices remains inconsistent. Therefore, we aimed to perform a systematic review and meta-analysis of the literature regarding the value of SMA Doppler indices for the early detection of neonates who are at increased risk of developing NEC.

## Methods

A pre-specified search protocol was formulated by two authors (D.R., pediatrician-neonatologist, with 10 years of experience and P.M., resident in pediatric surgery, with 2 years of experience) to examine whether Doppler ultrasonography of the SMA can assist in the early prediction of NEC. The study protocol was registered in PROSPERO (international prospective register of systematic reviews) (CRD42022316568). We formulated eligibility criteria using the P (Populations), I (Intervention), C (Comparison), O (Outcome) worksheet and search strategy. In detail, the population consisted of neonates, the intervention was defined by the performance of SMA Doppler ultrasonography, comparisons were performed between neonates who developed NEC and counterparts who did not develop NEC and the outcome was the manifestation of NEC.

A series of clinical questions were formulated, including the following:

- *1. What is the Doppler ultrasonography difference on the first postnatal day between neonates who developed NEC in comparison to those who did not?*
- *2. What is the Doppler ultrasonography difference at disease onset between neonates with and without NEC?*

The questions were elucidated based on the results of the systematic literature search.

### Search strategy

The Preferred Reporting Items for Systematic reviews and Meta-Analyses (PRISMA) guidelines were adopted for this systematic review. Two authors (D.R. and P.M.) independently performed a literature search in PubMed, Google Scholar and ScienceDirect. Disagreements were resolved by consensus or after consultation with a third reviewer (E.S., a pediatric gastroenterologist with15 years of experience). The literature was searched from 1 January 1990 to 31 January 2022, with filters [‘humans’] and search terms ‘necrotizing enterocolitis’ OR ‘NEC’ plus Boolean operator ‘AND,’ ‘superior mesenteric artery’ OR ‘SMA’ plus Boolean operator ‘AND,’ ‘Doppler’ or ‘ultrasound’ or ‘sonography.’ Additionally, the references of the identified studies were searched to ensure that no study was missed.

### Eligibility criteria and study selection

Eligible studies were those that included humans, with a full text available and published in English and with available neonatal data regarding Doppler ultrasonography indices (peak systolic velocity, end-diastolic velocity, time average mean velocity, the differential velocity that was calculated as peak systolic velocity-end-diastolic velocity [12], pulsatility index (PI) or resistive index). We included studies reporting the above data in neonates who developed NEC in comparison to those without NEC. Prospective, retrospective, longitudinal and cross-sectional studies were included. Review articles and opinion articles not reporting original data were excluded. A list of excluded studies was generated and reasons for exclusion were recorded.

### Extraction of data from selected studies: components of data extraction form

Two authors (D.R. and P.M.) independently reviewed the title, abstract and full text of the included studies. Data were extracted independently by the two reviewers on a Microsoft Excel spreadsheet using a predefined checklist. Extracted data included but were not limited to the following: author name, year, journal name, the country where the study was conducted, study design, study population, sample size and details of main findings, such as the differences between the subgroups of subjects regarding the Doppler ultrasonography, the risk of NEC and the risk of feeding intolerance.

### Quality assessment

Two authors (D.R. and P.M.) independently assessed the risk of bias using the Newcastle–Ottawa Scale for evaluating the methodological quality of studies, which is composed of three factors: selection, comparability and outcome/exposure [14]. For cohort studies, selection criteria assessed for the representativeness of the exposed cohort, the selection of the non-exposed cohort, the ascertainment of exposure and the demonstration that the outcome of interest was not present at the start of the study. For case-control studies, selection criteria were assessed for the representativeness of the cases, the selection of controls, the definition of cases and the definition of controls. The comparability of cohort and case-control studies was assessed based on the study design and the analysis of results including adjustment for potential confounding factors. For cohort studies we recorded ascertainment of the outcome, the follow-up period and the cohort retention, whereas, for case-control studies, we recorded ascertainment of exposure, the method of ascertainment for cases and controls and the non-response rate. We rated the quality of the studies (good, fair or poor) by awarding stars in each domain following the guidelines of the Newcastle–Ottawa Scale [14]. A “good” quality score required 3 or 4 stars in the selection, 1 or 2 stars in the comparability and 2 or 3 stars in the outcome/exposure domains. A “fair” quality score required 2 stars in the selection, 1 or 2 stars in the comparability and 2 or 3 stars in the outcome/exposure domain. A “poor” quality score reflected 0 or 1 star(s) in the selection, 0 stars in the comparability or 0 or 1 star(s) in the outcome/exposure domains.

### Analysis

Selected studies were subdivided into two groups: (a) studies that evaluated Doppler ultrasonography indices on the first postnatal day when neonates had no obvious signs or symptoms of NEC; (b) studies that evaluated Doppler ultrasonography indices at the onset of NEC.

Analyses were performed with Review Manager 5.4 (Review Manager (RevMan) Version 5.4, The Cochrane Collaboration, 2020, London, UK). In instances where continuous data outcome measures were not presented with their corresponding standard deviation but as a median (interquartile range), a standard deviation was calculated from the available interquartile range according to the *Cochrane Handbook for Systematic Review of Interventions* [15]. The weighted standardized mean differences of the SMA Doppler indices were calculated separately for the subgroups of neonates. The measurements were performed using a random-effects model, as described by DerSimonian et al. [16]. This model allows for inter-study variation and was chosen because heterogeneous populations were included in analyses. Heterogeneity was explored as the ratio between total heterogeneity and total variability with the *I*^2^ statistic. *I*^2^ can differentiate between true heterogeneity and sampling variance [17]. The standardized mean differences in SMA Doppler indices between neonatal subgroups were considered statistically significant at *P*<0.05. For the evaluation of publication bias, we created and examined a funnel plot.

## Results

### Study characteristics

**Fig. 1:**
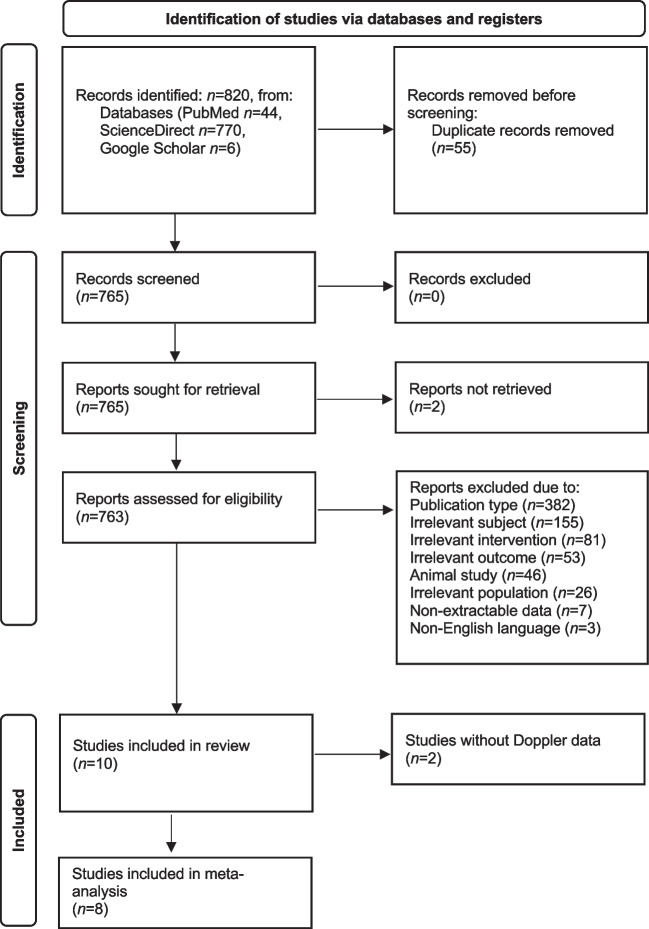
Preferred Reporting Items for Systematic reviews and Meta-Analyses flow diagram for study selection

The literature search identified 820 studies (44 in PubMed, 6 in Google Scholar and 770 in ScienceDirect). After scanning the titles, we excluded 55 duplicate studies. From the remaining 765 studies, we excluded two studies as the full text was not available. After screening titles, subjects and abstracts, 382 studies were excluded due to irrelevant study type, 155 studies due to irrelevant subject, 81 due to irrelevant intervention, 53 due to irrelevant outcome, 46 due to examining non-human subjects, 26 due to irrelevant population, 7 due to non-extractable data and 3 studies due to non-English language. Of the remaining 10 studies eligible for the review analysis [11–13, 18–24], 2 were not included in the final meta-analysis since one study did not provide data on Doppler ultrasound but instead, the odds ratio of developing NEC [23], whereas one study provided data on high-risk neonates but no data on the neonates who developed NEC in comparison to non-NEC neonates [19]. Thus, 8 studies were included in the final meta-analysis, as outlined in the PRISMA flow diagram (Fig. 1).

Among the eight studies included in the analysis (Tables 1 and 2), five were prospective case-control studies [11, 13, 20, 22, 24], whereas three were prospective cohort studies [12, 18, 21]. All studies were published between 1992 and 2019. Of the eight studies, two were performed in the UK [13, 20], two in Egypt [18, 21], and one each in China [12], Germany [11] India [22] and Lithuania [24]. Overall, a total of 494 neonates were included, among whom 126 developed NEC, while 368 neonates comprised the control group. Neonates in four studies were both term and preterm [11, 13, 20, 24], whereas four studies only examined preterm neonates [12, 18, 21, 22]. In four studies [11, 20–22], there were no differences in gestational age or birth weight between the neonatal groups examined; in two studies [13, 18], gestational age was similar, but birth weight was significantly lower in neonates with NEC compared to those without NEC; two studies [12, 24] did not report *P*-values for either gestational age or birth weight. Furthermore, two studies [12, 22] reported no difference in the incidence of patent ductus arteriosus, whereas the remaining six studies [11, 13, 18, 20, 21, 24] provided no data regarding the hemodynamic parameters between the aforementioned groups of neonates.

**Table 1:** Characteristics of the included studies examining Doppler indices on the first postnatal day

Study	Year	Country	Design	Population	Groups	SMA Doppler indices	NEC definition	NEC time onset	Results
Coombs et al. [20]	1992	UK	Prospective case–control	59 neonates: 27 term/preterm at-risk neonates (small for gestation age/low Apgar/exchange transfusion) 18 preterm controls 14 term controls	NEC: 3 (stages not provided) Controls: 56	Peak systolic velocity	Not provided	1, 7 and 21 days	Infants who developed NEC had a lower mean peak systolic velocity in comparison to non-NEC infants
Guang et al. [12]	2019	China	Prospective cohort	104 preterm neonates	NEC: 8 (2 stage I, 6 stage II) Controls: 96	Peak systolic velocity, end-diastolic velocity, differential velocity, time average mean velocity, PI, resistive index	Bell’s staging criteria as modified by Kliegman and Walsh	Median 12 (7, 14) days	A higher peak systolic velocity 54.165 (42.423–68.463) versus 42.195 (34.278–48.553) cm/s; (*P*=0.027) and differential velocity 47.445 (35.010–60.043) versus 32.565 (27.545–39.073) cm/s; (*P*=0.02) were significantly related to the risk of NEC
Khodair et al. [18]	2014	Egypt	Prospective cohort	52 preterm neonates	NEC: 12 (5 stage I, 6 stage II, 1 stage III) Controls: 40	Peak systolic velocity, end-diastolic velocity, PI, resistive index	Bell’s staging criteria as modified by Kliegman and Walsh	Not provided	Doppler indices of the SMA, peak systolic velocity (88.9 ± 17 cm/s and 53 ± 8.5 cm/s), end-diastolic velocity (18.75 ± 11.3 cm/s and 14.9 ± 5.6 cm/s), resistive index (0.78 ± 0.09 and 0.67 ± 0.1) and PI (1.53 ± 0.73 and 0.67 ± 0.15) were higher in NEC group than in controls, with statistically significant differences
Louis et al. [22]	2013	India	Prospective case–control	100 preterm intrauterine growth restriction/small for gestation age neonates 50 neonates with absent/reversed end-diastolic flow 50 controls with normal Doppler	In at-risk population: NEC: 16 (10 stage I, 6 stage II) In controls: NEC: 2 (1 stage I, 1 stage II)	End-diastolic velocity, resistive index	Bell’s staging criteria as modified by Kliegman and Walsh	Median 14 (3, 32) h	Resistive index on day 1 was higher in babies with absent/reversed end-diastolic flow [5.4 (3.3, 7.3)] who developed NEC compared to the control group [3.3 (1.7, 3.9)], (*P*=0.049). On further analysis, within absent/reversed end-diastolic flow group, end-diastolic velocity on day 1 (− 3.3 ± 4.1 vs. − 0.6 ± 5.1 cm/s; *P*=0.03) was lower in the neonates with NEC compared to those without NEC

*NEC* necrotizing enterocolitis, *PI* pulsatility index, *SMA* superior mesenteric artery

**Table 2:** Characteristics of the included studies examining Doppler indices on NEC onset

Study	Year	Country	Design	Population	Groups	SMA Doppler indices	NEC definition	NEC time onset	Results
Deeg et al. [11]	1993	Germany	Prospective case-control study	28 neonates: 14 term/preterm neonates with NEC 14 term/preterm controls	NEC: 14 (stages not provided) Controls: 14	Peak systolic velocity, end-diastolic velocity, time average mean velocity, resistive index	Not provided	Mean 15 ± 10 days	In the SMA of patients with NEC, the peak systolic velocity was 119.0 ± 57.7 cm/s, the end-diastolic velocity 10.2 ± 8.9 cm/s, and the time average mean velocity 28.0 ± 13.3 cm/s. The resistive index in patients with NEC was 0.88 ± 0.12. In controls, the following flow velocities were measured in the SMA: peak systolic velocity 68.4 ± 20.5 cm/s, end-diastolic velocity 11.8 ± 6.8 cm/s and time average mean velocity 13.0 ± 5.5 cm/s. The resistive index in the SMA of healthy infants was 0.84 ± 0.08. Statistical comparison with the healthy control group showed a significant increase in the peak systolic velocity and time average mean velocity, with a *P*<0.05 in patients with NEC
Hashem et al. [21]	2017	Egypt	Prospective cohort study	51 septic preterm neonates: 25 neonates with NEC 26 neonates with sepsis but no NEC	NEC: 25 (21 stage I, 3 stage II, 1 stage III) Controls: 26	Peak systolic velocity, end-diastolic velocity, PI, resistive index	Bell’s staging criteria as modified by Kliegman and Walsh	Median 12 days	A statistically significant lower peak systolic velocity was found (*P*=0.001) and a lower end-diastolic velocity (*P*=0.001) in the SMA in the group with clinical signs of NEC in comparison with the group with no clinical signs of NEC
Kempley et al. [13]	1992	UK	Prospective case–control study	38 neonates: 19 preterm neonates with NEC 19 term/preterm controls	NEC: 19 (10 stage I, 9 stage II) Controls: 19	Time average mean velocity, PI	British Association of Perinatal Medicine criteria	Mean 15 ± 13.4 days	Mean SMA velocity was significantly higher in the infants with confirmed NEC (36.5 cm/s) than in infants with suspected, unconfirmed disease (19.6 cm/s, *P*<0.05)
Urboniene et al. [24]	2015	Lithuania	Prospective case-control study	62 neonates: 29 term/preterm neonates with NEC 33 term/preterm controls	NEC: 29 (13 stage I, 12 stage II, 4 stage 3) Controls: 33	Peak systolic velocity, time average mean velocity, PI, resistive index	Bell’s staging criteria as modified by Kliegman and Walsh	Not provided	The differences in the Doppler indices of SMA including peak systolic velocity, and time average mean velocity were not statistically significant between NEC and the control group. However, the values of resistive index and PI of SMA were significantly different between NEC and control groups (*P*<0.001)

*NEC* necrotizing enterocolitis, *PI* pulsatility index, *SMA* superior mesenteric artery

Of the eight studies, four examined Doppler ultrasound indices during the first postnatal day in high-risk neonates [12, 18, 20, 22],  one examined neonates born small for gestational age, with low Apgar, or having undergone exchange transfusion in comparison to controls, reporting that all three neonates who developed NEC belonged in the at-risk group [20]; of them, the first neonate who was severely growth-restricted developed NEC on the first day of life, the second neonate on the seventh and the third neonate in the third week of life [20]. A second study evaluated neonates with abnormal in comparison to those with normal umbilical artery Doppler indices, reporting that 32% of the neonates in the absent or reversed end-diastolic flow group developed NEC (20% developed NEC IA and 12% NEC IIA), compared to 4% of the neonates in the control group (2% developed each NEC IA and IIA) [22]. In that study, 75% of NEC cases in the absent or reversed end-diastolic flow group occurred within the first 24 h, even before starting feeds, whereas in non-absent or reversed end-diastolic flow neonates, NEC was developed at a significantly later time (median 155 h of life) [22]. The two remaining studies examined cohorts of preterm neonates [12, 18]; one study reported that the eight neonates who developed NEC (two grade I and six grade II) were more likely to proceed to antenatal steroid therapy, although there were no significant differences in other characteristics, compared to neonates without NEC [12], and one study reported that between the 12 neonates who developed NEC (five of them with grade I NEC, six with grade II and one with grade III) and the non-NEC group, there were no significant differences in gestational age, birth weight or sex [18]. Of the four studies that examined Doppler ultrasound indices following onset of NEC [11, 13, 21, 24],  in two [11, 13], the onset of NEC was on the 15^th^ postnatal day, in one on the 12^th^ postnatal day [21], whereas in one study [24] the time of the NEC manifestation was not provided. Necrotizing enterocolitis was defined according to the staging criteria of Bell et al. as modified by Kliegman and Walsh in five studies [12, 18, 21, 22, 24] and the British Association of Perinatal Medicine criteria in two [13], whereas in two studies, although the authors provided clinical and radiological features of the neonates with NEC, the specific criteria used for the diagnosis of NEC were not provided [11, 20]. In six studies, the authors included cases of both suspected and confirmed NEC [12, 13, 18, 21, 22, 24], whereas, in two studies, the diagnostic certainty of NEC was not provided [11, 20]. With regard to the Doppler ultrasound indices, peak systolic velocity was recorded in five studies [11, 12, 18, 20, 21], end-diastolic velocity in five [11, 12, 18, 21, 22], time average mean velocity in three [11–13], differential velocity in one [12], PI in four [12, 13, 18, 21] and resistive index in six studies [11, 12, 18, 21, 22]. The acquisition of SMA Doppler indices was performed with a linear [11, 12, 18, 24], curvilinear [21] or short focus probe [20], in sagittal (longitudinal) plane [11, 12, 18, 20–22], detecting the SMA in the epigastrium below the xiphisternum [12, 20–22]. Among the four studies examining Doppler ultrasound indices during the first postnatal day, in two studies [12, 22], SMA Doppler was performed before the initiation of feeding, in one [20] before feeding in all high-risk, in all but one preterm control and in almost half of term control neonates, whereas one study did not provide this information [18].

The quality of studies was assessed according to the Newcastle-Ottawa scale. Table 3 depicts the scores given to each study. All studies were rated as “good quality”. Declarations of funding or conflicts of interest were reported as “none” in three studies [12, 21, 22], whereas five studies [11, 13, 18, 20, 24] did not provide this information.

**Table 3:** Assessment of the quality of studies utilizing the Newcastle-Ottawa Quality Assessment Scale for case-control and cohort studies

	Case-control studies								
	Selection				Comparability	Outcome / Exposure			Quality score
	Adequate definition of cases	Representativeness of the cases	Selection of controls	Definition of controls	Comparability	Assessment of exposure	Assessment method	Non-response rate	
Coombs et al. [20]	*	*	-	*	*	*	*	-	Good
Deeg et al. [11]	*	*	-	*	*	*	*	-	Good
Kempley et al. [13]	*	*	-	*	*	*	*	-	Good
Louis et al. [22]	*	*	-	*	**	*	*	-	Good
Urboniene et al. [24]	*	*	-	*	*	*	*	-	Good
	Cohort studies								
	Assessment of exposure	Representativeness of the exposed cohort	Selection of non-exposed cohort	Outcome of interest was not present at start	Comparability	Assessment of outcome	Enough follow-up	Adequacy of follow-up	Quality score
Guang et al. [12]	*	-	*	*	*	*	*	*	Good
Hashem et al. [21]	*	-	*	*	*	*	*	*	Good
Khodair et al. [18]	*	-	*	*	*	*	*	*	Good

Good quality: 3 or 4 stars (*) in the selection domain AND 1 or 2 stars in the comparability domain AND 2 or 3 stars in the outcome domain. Fair quality: 2 stars in the selection domain AND 1 or 2 stars in the comparability domain AND 2 or 3 stars in outcome/exposure domain. Poor quality: 0 or 1 star in selection domain OR 0 stars in comparability domain OR 0 or 1 star in outcome/exposure domain

### Publication bias

**Fig. 2:**
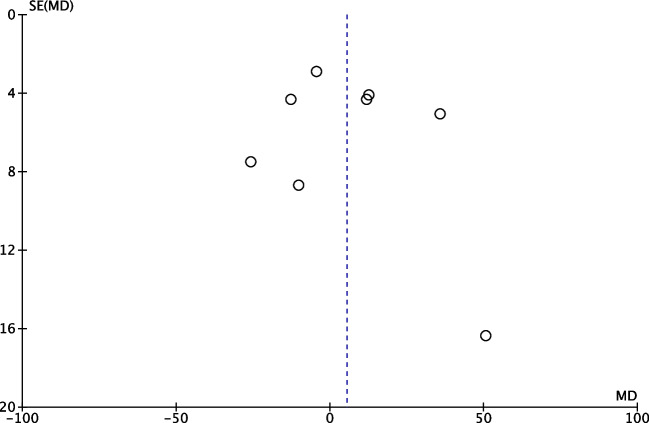
Funnel plot of the included studies. In the figure, the x-axis represents the magnitude of the effect and the y-axis the “precision.” The precision estimate used is the standard error. The broken line indicates the estimated common effect of the meta-analysis (i.e. the mean difference). *MD* mean difference, *SE* standard error

Publication bias was examined using a funnel plot (Fig. 2). From the visual inspection of the funnel plot created, there is low suspicion of publication bias.

- *1. What is the Doppler ultrasonography difference on the first postnatal day between neonates who developed NEC in comparison to those who did not?*

**Fig. 3:**
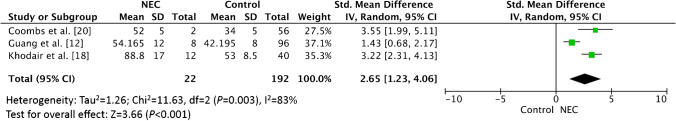
Forest plot of the standardized mean difference of the peak systolic velocity on the first postnatal day for neonates who developed necrotizing enterocolitis (NEC) compared to those who did not*. CI* confidence intervals, *IV* inverse variance, *SD* standard deviation

Among the four studies that examined Doppler ultrasound indices during the first postnatal day, the authors of three [12, 18, 20] suggested that an increased peak systolic velocity was recorded in neonates who developed NEC. Guang et al. [12] and Khodair et al. [18] reported that a significantly higher peak systolic velocity was recorded in neonates who developed NEC compared to controls, whereas Coombs et al. [20] reported that among the three neonates who developed NEC, the peak systolic velocity was elevated in the first, reduced in the second and was normal in the third neonate with NEC in comparison to non-NEC neonates. In the meta-analysis of those three studies, we found a significantly higher peak systolic velocity in neonates who developed NEC compared to those who did not, with a mean difference of 2.65 cm/s [95% confidence intervals (CI) 1.23, 4.06, overall effect *Z*=3.66, *P*<0.001], with heterogeneity *I*^2^=83% (Fig. 3).

Guang et al. [12] and Khodair et al. [18] reported that end-diastolic velocity was higher in neonates with NEC in comparison to non-NEC neonates, whereas Louis et al. [22] recorded that among neonates with absent or reversed end-diastolic flow, end-diastolic velocity was lower in the neonates with NEC compared to those without. The meta-analysis of these three studies suggested a non-significant difference (*Z*=0.14, *P*=0.89) in end-diastolic velocity between the two groups of 0.05 cm/s (95% CI − 0.60, 0.70), with heterogeneity *I*^2^=66% (Supplemental Fig. 1).

The time average mean velocity and differential velocity were reported in only one study [12]; therefore, no meta-analysis was performed.

**Fig. 4:**
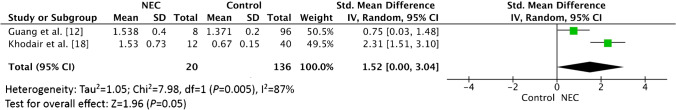
Forest plot of the standardized mean difference of the pulsatility index on the first postnatal day for neonates who developed necrotizing enterocolitis (NEC) compared to those who did not. *CI* confidence intervals, *IV* inverse variance, *SD* standard deviation

The studies by Guang et al. [12] and Khodair et al. [18] provided data regarding the PI, reporting that PI was higher in neonates who developed NEC compared to controls. The meta-analysis of the two studies suggested a higher PI in neonates who developed NEC with a mean difference of 1.52 (95% CI 0.00, 3.04, *Z*=1.96, *P*=0.05) compared to those who did not, with heterogeneity *I*^2^=87% (Fig. 4).

**Fig. 5:**
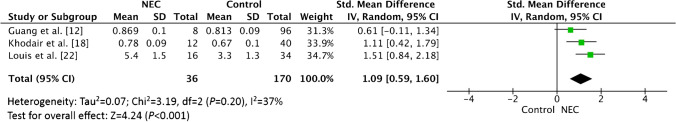
Forest plot of the standardized mean difference of the resistive index on the first postnatal day in neonates who developed necrotizing enterocolitis (NEC) compared to those who did not*. CI* confidence intervals, *IV* inverse variance, *SD* standard deviation

Finally, three studies by Guang et al. [12], Khodair et al. [18] and Louis et al. [22] reported that the resistive index was higher in neonates with NEC in comparison to non-NEC neonates. The meta-analysis of the three studies suggested a significantly higher resistive index in neonates who developed NEC with a mean difference of 1.09 (95% CI 0.59, 1.60, *Z* = 4.24, *P* < 0.001) between the two groups, with heterogeneity *I*^2^ = 37% (Fig. 5).

- *2. What is the Doppler ultrasonography difference at disease onset between neonates with and without NEC?*

Of the four studies that examined Doppler indices at the onset of NEC, three [11, 21, 24] reported data for peak systolic velocity. Deeg et al. [11] reported a significant increase in peak systolic velocity in patients with NEC in comparison to healthy controls. In contrast, Hashem et al. [21] reported a significantly lower peak systolic velocity in the septic group with clinical signs of NEC in comparison to the septic group with no clinical signs of NEC, whereas Urboniene et al. [24] reported no significant difference in peak systolic velocity between NEC and control groups. Meta-analysis of these three studies suggested no significant mean difference (*Z*=0.18, *P*=0.86) in peak systolic velocity of  − 0.10 cm/s (95% CI − 1.13, 0.94) between the two groups, with heterogeneity *I*^2^=88% (Supplemental Fig. 2).

Deeg et al. [11] and Hashem et al. [21] reported a lower end-diastolic velocity in neonates with NEC in comparison to neonates without NEC. Meta-analysis of these two studies suggested no significant mean difference (*Z*=1.56, *P*=0.12) in end-diastolic velocity of  − 0.64 cm/s (95% CI − 1.45, 0.17) between the two groups, with heterogeneity *I*^2^=66% (Supplemental Fig. 3).

In three studies, the time average mean velocity in neonates with NEC compared to those without was reported [11, 13, 24]. Deeg et al. [11] and Kempley et al. [13] found a higher time average mean velocity in neonates with NEC as compared to controls, whereas Urboniene et al. [24] reported that time average mean velocity was lower, although non-significant, in neonates with NEC compared to those without. The meta-analysis of the three studies suggested no significant mean difference (*Z*=1.35, *P*=0.18) in time average mean velocity of 8.26 cm/s (95% CI − 3.72, 20.24) between the two groups, with heterogeneity *I*^2^=91% (Supplemental Fig. 4).

The study by Hashem et al. [21] reported that PI was higher in neonates with NEC compared to neonates without, although the difference was not significant. In contrast, Kempley et al. [13] and Urboniene et al. [24] reported that PI was lower in neonates who developed NEC as compared to controls. The meta-analysis of the three studies suggested no significant mean difference (*Z*=1.13, *P*=0.26) of  − 0.63 (95% CI − 1.72, 0.46) between neonates who developed NEC compared to those who did not, with heterogeneity *I*^2^=90% (Supplemental Fig. 5).

Finally, the studies of Deeg et al. [11] and Hashem et al. [21] reported that the resistive index was higher in neonates with NEC in comparison to those without. However, Urboniene et al. [24] reported a significantly lower resistive index in neonates with NEC compared to controls. The meta-analysis of the three studies suggested no significant mean differnce (*Z*=0.92, *P*=0.36) in resistive index of  − 1.19 (95% CI − 3.72, 1.34) between the two groups, with heterogeneity *I*^2^=97% (Supplemental Fig. 6).

## Discussion

Our findings suggest that SMA Doppler indices during the first postnatal day, in particular peak systolic velocity, PI and resistive index, are associated with the subsequent development of NEC. On the other hand, our meta-analysis findings do not support a strong association between Doppler ultrasound indices with the development of NEC at the time of disease onset.

The etiology of NEC is complex and multifactorial, including genetic predisposition, intestinal immaturity, deranged vascular tone, intestinal ischemia, abnormal microbial colonization and highly immunoreactive intestinal mucosa [1, 25]. The process of ischemia followed by reperfusion has a significant impact on the newborn intestinal endothelium, significantly compromising the constitutive and stimulated production of nitric oxide [26, 27]. This ischemia-reperfusion-induced loss of endothelial nitric oxide production has been associated with a pronounced and sustained intestinal ischemia that increases the risk of the later development of NEC [28]. Neonates who are at increased risk of developing NEC are those with intrauterine growth restriction and absent or reversed end-diastolic flow [29]. These high-risk neonates almost always have fetal hemodynamic disturbances in the umbilical artery. The ischemia and hypoxemia produce circulatory redistribution toward the brain and away from the viscera, particularly the gastrointestinal system. The prolonged redistribution may cause structural, neuromotor, secretory and mucosal functional alterations of the intestinal tissue, so that postnatally, the intestine is more susceptible to dysmotility and abnormal bacterial colonization [19, 30]. To date, several studies have demonstrated a close association between absent or reversed end-diastolic flow and NEC, which appears to be independent of other variables such as the degree of growth retardation, prematurity and perinatal asphyxia [4, 31–42]. Of the studies included in our review that examined the Doppler indices during the first postnatal day, two (Coombs et al. [20] and Louis et al. [22]) examined the postnatal Doppler indices in high-risk neonates defined by absent or reversed end-diastolic flow, small for gestational age or low Apgar in comparison to controls, whereas two (Guang et al. [12] and Khodair et al. [18]) enrolled cohorts of preterm neonates. Risk factors, and especially absent or reversed end-diastolic flow of the umbilical artery, were strongly implicated in the development of NEC, as suggested by Louis et al., where a significantly higher number of neonates with absent or reversed end-diastolic flow developed NEC, mainly within the first 24 h, compared to neonates with normal umbilical artery Doppler indices [22] and by Coombs et al., where all three neonates with NEC were in the high-risk group [20]. Evidence of absent or reversed end-diastolic flow in the antenatal Doppler evaluation of the umbilical artery has been associated with significant overall odds for developing NEC, as demonstrated by Dorling et al. [42]; and thus, the Doppler assessment of the umbilical artery in high-risk pregnancies might help in assessing the severity of fetal decompensation and the postnatal risk of NEC [42]. Furthermore, our findings support the notion that in high-risk neonates, the postnatal differences in SMA Doppler indices reveal the continuation of redistribution which started prenatally [19, 30]. In particular, the elevated peak systolic velocity, PI and resistive index indicate vasoconstriction in the SMA that may suggest profound bowel ischemia. Nevertheless, in one study not included in our meta-analysis, Murdoch et al. [23] (who examined the SMA Doppler indices in 64 term and preterm neonates), suggested that when adjusted for gestational age at birth, end-diastolic velocity, time average mean velocity and PI were significantly predictive of the risk of NEC. In addition, Bora et al. [19] examined the SMA Doppler indices in 62 term and preterm neonates (23 small for gestational age with absent or reversed end-diastolic flow in the umbilical artery, 20 small for gestational age with normal umbilical artery Dopplers and 19 appropriate for gestational age with normal umbilical artery Dopplers) reporting that time average mean velocity was lower in the first and second groups compared to the third group, whereas peak systolic velocity was significantly lower in the first compared to the third group; however, no data on the neonates who developed NEC were provided in comparison to non-NEC neonates. Furthermore, in animal models, it was found that an insult in susceptible animals may lead to early profound bowel ischemia, which may trigger NEC [8]. Among several factors acting in concert to cause the development of NEC, the roles of gestational age, birth weight and hemodynamically significant patent ductus arteriosus have been established [20, 43]. In this meta-analysis, most studies were matched for gestational age and birth weight; however, data regarding hemodynamic parameters and patent ductus arteriosus were not provided. Although changes in perfusion may not be the sole factor, abnormalities either of the development of the splanchnic circulation or vasoconstriction of the circulation in neonatal life play a role in the etiology of NEC [8, 9]; and thus, the evaluation of SMA flowmetry parameters during the first day of life may be useful at least for high-risk neonates who are at risk of developing NEC.

On the other hand, the findings of this meta-analysis do not support a strong association between Doppler ultrasound indices and the development of NEC at the time of disease onset. Deeg et al. reported that peak systolic velocity, PI and resistive index in SMA Doppler were elevated in neonates with NEC compared to those without [11], whereas Hashem et al. found a lower peak systolic velocity but higher PI and resistive index in neonates with NEC compared to neonates without NEC [21], and finally, two studies (Kempley et al. [13] and Urboniene et al. [24]) found all parameters (peak systolic velocity, PI and resistive index) were lower in neonates with NEC compared to neonates without NEC. These discrepancies might be explained by a specific perfusion pattern that exists in the manifestation of NEC. Previous studies have suggested that the pattern of splanchnic resistance among infants with NEC may be biphasic with an initial high resistance preceding the development of the condition [13, 23]. This may be followed by low resistance because of the effects of inflammation and sepsis as the condition progresses [13]. This may reflect an infective and inflammatory component in active NEC characterized by splanchnic hyperemia [44]. Therefore, based on the phase of the disease during which Doppler is performed, ultrasonographic indices may reveal that neonates are either in the early stages of NEC with evidence of splanchnic ischemia or in the later advanced stages with evidence of splanchnic hyperemia, possibly with some element of vasoconstriction [23]. Given that neonates with both early and advanced NEC were examined in the studies included in our systematic review and meta-analysis, we cannot be certain that the included neonates had been evaluated in any of the ischemia or hyperemia phases of SMA perfusion.

### Limitations and areas in need of future research

The current review is based on a comprehensive evaluation of the current literature, including studies that have examined the SMA Doppler indices on the first postnatal day and at disease onset. All studies were of good methodological quality; however, the degree of heterogeneity when examining all parameters, even in subgroup analyses, was high. An explanation for the high degree of heterogeneity could be that the number of studies, as well as the total number of neonates included, was relatively small [45]. Furthermore, there were differences in the design of the studies included. Of note, there was an inconsistency in terms of the number of sonographers who performed the Doppler ultrasound scans, as there were studies in which the same sonographer performed all examinations, while in others multiple sonographers performed the Doppler ultrasounds. Additional differentiating factors include the number of measurements, the duration of the measurements and the timing of measurements, even within the same subgroups. Furthermore, not all studies acquired the same Doppler indices, which renders the complete examination of the intervention difficult. Although most included studies were matched for gestational age and birth weight, six out of eight studies provided no data regarding hemodynamic parameters or patent ductus arteriosus. Finally, there was a difference in the definition and staging of NEC, as some authors implemented Bell’s staging criteria, while others opted for the British Association of Perinatal Medicine criteria.

The early detection of neonates at high risk of developing NEC is important in clinical practice, and Doppler ultrasound should be utilized in detecting changes in intestinal perfusion before severe damage to the intestinal epithelium occurs. Future well-designed studies are warranted to evaluate the cutoff limits of the Doppler ultrasound indices before and during NEC manifestation.

## Conclusions

This meta-analysis suggests that SMA Doppler parameters during the first postnatal day, namely peak systolic velocity, PI and resistive index, are higher in neonates with NEC compared to those without. On the other hand, once NEC has developed, the evaluation of SMA Doppler indices may not be conclusive since the volumetric indices are different in the ischemic and hyperemic phases of the disease.

## Supplementary Information

Below is the link to the electronic supplementary material.

Supplementary file1 (PDF 797 KB)

Supplementary file2 (PDF 812 KB)

Supplementary file3 (PDF 734 KB)

Supplementary file4 (PDF 816 KB)

Supplementary file5 (PDF 809 KB)

Supplementary file6 (PDF 834 KB)

## Data Availability

The datasets analyzed during the current study are available from the corresponding author on reasonable request.
