# Supplementary figures and images for: A systematic review and meta-analysis of the role of Doppler ultrasonography of the superior mesenteric artery in detecting neonates at risk of necrotizing enterocolitis
Source: Pediatr Radiol. 2023 Jun 13;53(10):1989–2003. doi: 10.1007/s00247-023-05695-6 (PMC10497699; doi:10.1007/s00247-023-05695-6)

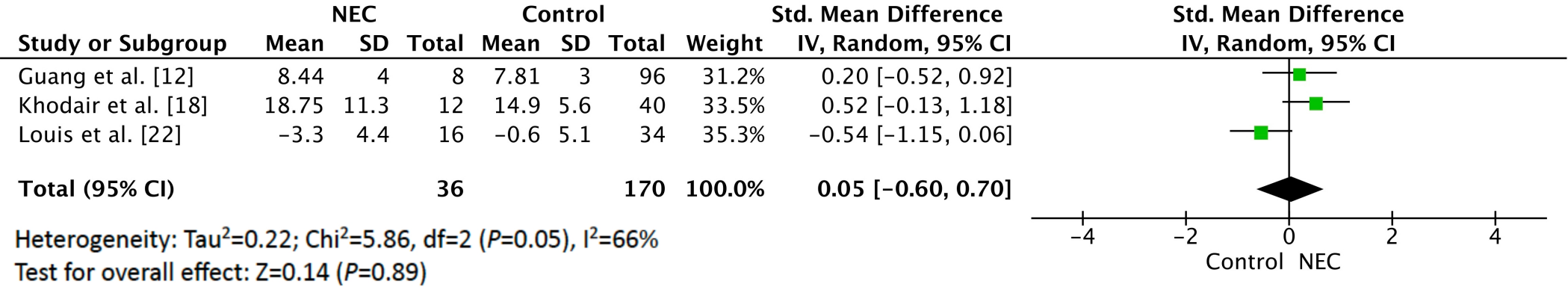

Supplement: Supplementary file 1 — Supplementary file1 (PDF 797 KB) [file 247_2023_5695_MOESM1_ESM.pdf]

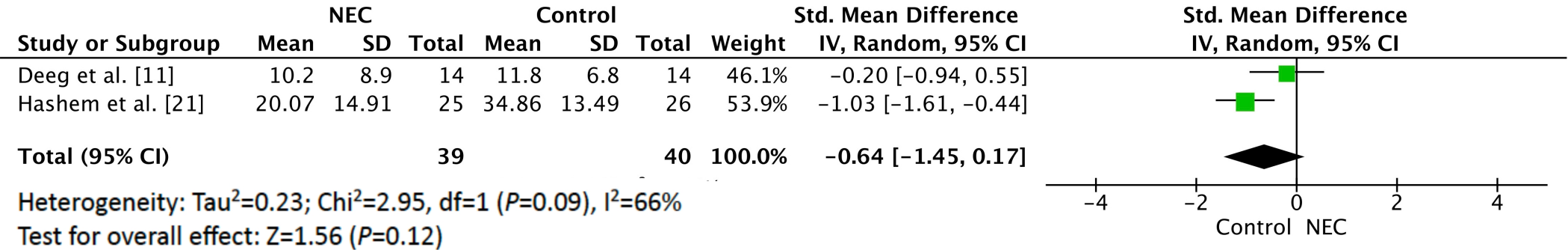

Supplement: Supplementary file 3 — Supplementary file3 (PDF 734 KB) [file 247_2023_5695_MOESM3_ESM.pdf]

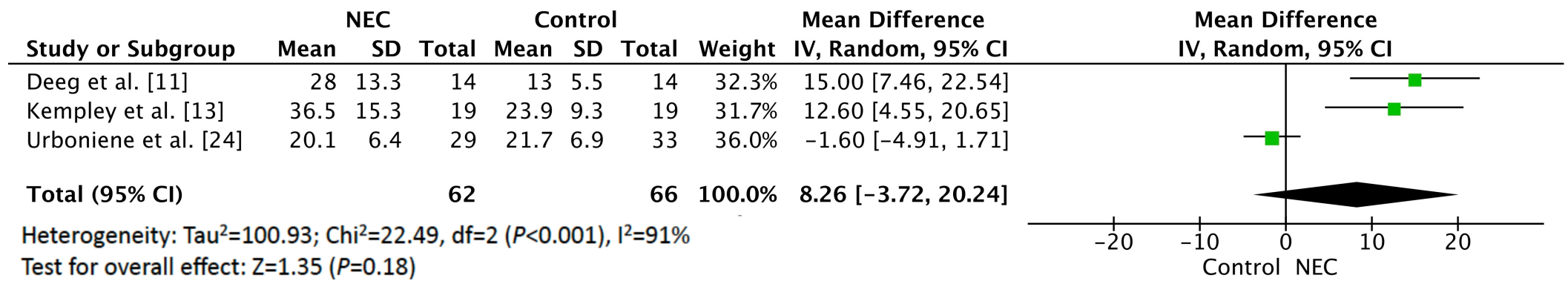

Supplement: Supplementary file 4 — Supplementary file4 (PDF 816 KB) [file 247_2023_5695_MOESM4_ESM.pdf]

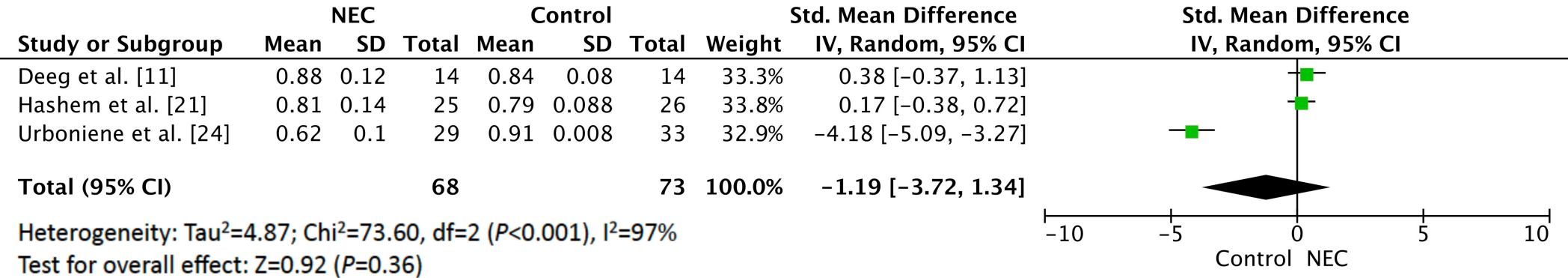

Supplement: Supplementary file 6 — Supplementary file6 (PDF 834 KB) [file 247_2023_5695_MOESM6_ESM.pdf]
