# Supplementary material for: A systematic review and meta-analysis of the role of Doppler ultrasonography of the superior mesenteric artery in detecting neonates at risk of necrotizing enterocolitis
Source: Pediatr Radiol. 2023 Jun 13;53(10):1989–2003. doi: 10.1007/s00247-023-05695-6 (PMC10497699; doi:10.1007/s00247-023-05695-6)

Study or Subgroup	NEC Mean	NEC SD	NEC Total	Control Mean	Control SD	Control Total	Weight	Std. Mean Difference IV, Random, 95% CI
Deeg et al. [11]	119	57.7	14	68.4	20.5	14	31.0%	1.13 [0.33, 1.94]
Hashem et al. [21]	47.01	27.05	25	72.72	26.54	26	34.0%	−0.94 [−1.53, −0.36]
Urboniene et al. [24]	34.2	10.6	29	38.4	12.2	33	35.0%	−0.36 [−0.86, 0.14]
**Total (95% CI)**			**68**			**73**	**100.0%**	**−0.10 [−1.13, 0.94]**

Heterogeneity: Tau^2=0.74; Chi^2=16.92, df=2 (P<0.001), I^2=88%

Test for overall effect: Z=0.18 (P=0.86)

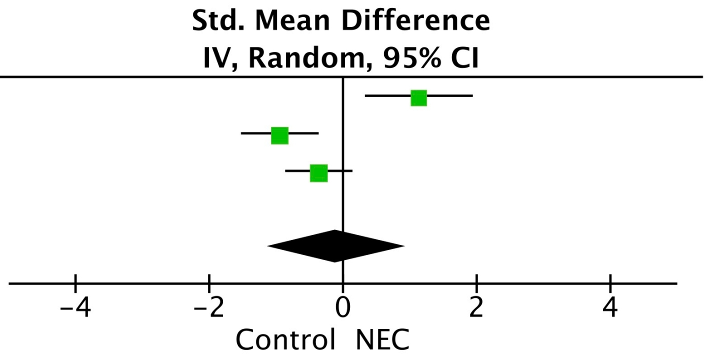

Supplement: Supplementary file 2 — Supplementary file2 (PDF 812 KB) [file 247_2023_5695_MOESM2_ESM.pdf]
