# Supplementary material for: A systematic review and meta-analysis of the role of Doppler ultrasonography of the superior mesenteric artery in detecting neonates at risk of necrotizing enterocolitis
Source: Pediatr Radiol. 2023 Jun 13;53(10):1989–2003. doi: 10.1007/s00247-023-05695-6 (PMC10497699; doi:10.1007/s00247-023-05695-6)
Supplement: Supplementary file 5 — Supplementary file5 (PDF 809 KB) [file 247_2023_5695_MOESM5_ESM.pdf]

| | NEC | | | Control | | | | Std. Mean Difference |
|---|---|---|---|---|---|---|---|---|
| Study or Subgroup | Mean | SD | Total | Mean | SD | Total | Weight | IV, Random, 95% CI |
| Hashem et al. [21] | 2.017 | 1.421 | 25 | 1.75 | 0.43 | 26 | 33.8% | 0.25 [−0.30, 0.80] |
| Kempley et al. [13] | 2.17 | 0.9 | 19 | 2.72 | 1 | 19 | 32.7% | −0.57 [−1.22, 0.08] |
| Urboniene et al. [24] | 1.66 | 0.41 | 29 | 2.69 | 0.79 | 33 | 33.5% | −1.59 [−2.16, −1.01] |
| **Total (95% CI)** | | | **73** | | | **78** | **100.0%** | **−0.63 [−1.72, 0.46]** |

**Std. Mean Difference**
**IV, Random, 95% CI**

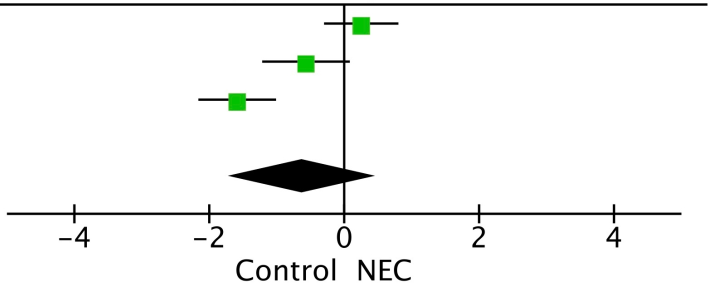


Heterogeneity: $Tau^2=0.84$; $Chi^2=20.44$, df=2 ($P<0.001$), $I^2=90\%$

Test for overall effect: Z=1.13 ($P=0.26$)
